# Cellular Compartmentalization, Glutathione Transport and Its Relevance in Some Pathologies

**DOI:** 10.3390/antiox12040834

**Published:** 2023-03-29

**Authors:** Héctor Vázquez-Meza, María Magdalena Vilchis-Landeros, Melissa Vázquez-Carrada, Daniel Uribe-Ramírez, Deyamira Matuz-Mares

**Affiliations:** 1 Departamento de Bioquímica, Facultad de Medicina, Universidad Nacional Autónoma de México, Avenida Universidad 3000, Cd. Universitaria, Coyoacán, Mexico City 04510, Mexico; hvazquez@bq.unam.mx (H.V.-M.); vilchisl@unam.mx (M.M.V.-L.); 2 Departamento de Microbiología, Instituto Politécnico Nacional, Escuela Nacional de Ciencias Biológicas, Plan de Carpio y Plan de Ayala S/N Santo Tomás, Miguel Hidalgo, Mexico City 11340, Mexico; melissa@bq.unam.mx; 3 Departamento de Ingeniería Bioquímica, Instituto Politécnico Nacional, Escuela Nacional de Ciencias Biológicas, Av. Wilfrido Massieu 399, Nueva Industrial Vallejo, Gustavo A. Madero, Mexico City 07738, Mexico; danielur@bq.unam.mx

**Keywords:** glutathione, S-glutathionylation, transporters, deficiency

## Abstract

Reduced glutathione (GSH) is the most abundant non-protein endogenous thiol. It is a ubiquitous molecule produced in most organs, but its synthesis is predominantly in the liver, the tissue in charge of storing and distributing it. GSH is involved in the detoxification of free radicals, peroxides and xenobiotics (drugs, pollutants, carcinogens, etc.), protects biological membranes from lipid peroxidation, and is an important regulator of cell homeostasis, since it participates in signaling redox, regulation of the synthesis and degradation of proteins (S-glutathionylation), signal transduction, various apoptotic processes, gene expression, cell proliferation, DNA and RNA synthesis, etc. GSH transport is a vital step in cellular homeostasis supported by the liver through providing extrahepatic organs (such as the kidney, lung, intestine, and brain, among others) with the said antioxidant. The wide range of functions within the cell in which glutathione is involved shows that glutathione’s role in cellular homeostasis goes beyond being a simple antioxidant agent; therefore, the importance of this tripeptide needs to be reassessed from a broader metabolic perspective.

## 1. Introduction

Oxidative stress is one of the main causes of the development of different types of diseases, such as cancer [1,2], neurodegenerative pathologies [3,4], liver [5,6], cardiac [7,8], pulmonary [9,10] and renal diseases [11,12]. Therefore, strategies have been developed to reduce its effects, such as modifying the lifestyle of patients, that is, changes in diet and physical activity; abolishing any habit that generates oxidizing molecules (such as smoking or drinking alcohol) is also important. With such measures, it is sought to strengthen the antioxidant systems of the patient, for prevention of disease or to decrease its effects [13,14,15,16].

Regarding oxidative stress, the enzymatic systems that contribute the most to the generation of ROS include the proteins that are bound to the plasma membrane, such as the family of NADPH oxidases [17,18], the enzymatic systems that participate in the lipid metabolism within peroxisomes and the activity of various cytosolic enzymes such as cyclooxygenases. Although all these sources contribute to the increase in the oxidative state of the cell, the vast majority of cellular ROS (approximately 90%) originates from the mitochondria [19,20].

**Figure 1:**
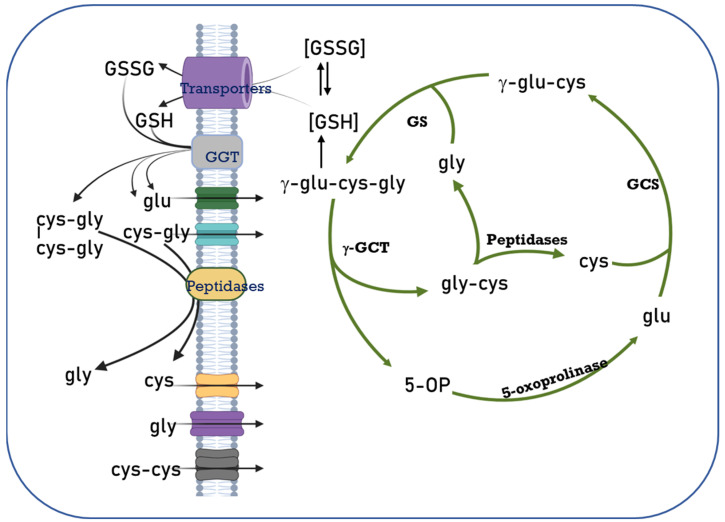
The Glutathione Cycle. Cellular glutathione homeostasis is directly related to its synthesis, degradation, transport, and the availability of the amino acids that make up the non-protein thiol. Different enzymes participate in this regulation process, such as γ-glutamylcysteine synthetase (GCS), glutathione synthetase (GS), 5-oxoprolinase, glutathione transporters, γ-glutamyl transpeptidases (GGT), membrane peptidases and amino acid transporters. 5-OP: 5 oxoproline. Modified from [30].

To counteract the effect of ROS, the cell has a series of antioxidant compounds. One of the most important antioxidant molecules in cellular systems is reduced glutathione (GSH). This tripeptide (glutamate, cysteine and glycine) [21,22] is the most abundant non-protein thiol in cells, with concentration reaching up to 15 mM [20]. Most of this glutathione is in a reduced state (about 99%), the remaining 1% being oxidized glutathione (GSSG) [23,24]. The concentration of glutathione is regulated by different processes, such as its own synthesis, its re-oxidation, its use for the detoxification of diverse substances (such as alcohol and drugs), and its transport to the different intracellular and extracellular compartments. (Figure 1) [25,26]. Glutathione, through the multiple activities and functions in which it participates (neutralization of free radicals, donor of reducing equivalents, coenzyme, elimination of xenobiotics and other endogenous metabolites, etc.), is important for cellular homeostasis, since it is involved in the dynamic balance that the organism requires for its proper functioning and morphological integrity [27,28,29].

In this review, we describe the importance of glutathione, both inside and outside of the cell, its transport, its cellular compartmentalization and some associated deficiency diseases.

## 2. Glutathione Intracellular Compartmentalization

The conservation of hepatic glutathione levels is a dynamic process resulting from the balance between the synthesis rate, transport, use and removal of such thiols [26]. Its synthesis takes place only in the cellular cytosol since all the necessary enzymes for its synthesis are found there [31]. Nevertheless, within the cell, glutathione is compartmentalized into different cell organelles and ratios. A concentration of 1–15 mM is found in the cell cytosol [20,29]. GSH is also present in the endoplasmic reticulum, nuclear matrix and peroxisomes, but at concentrations that need to be determined [27,32].

Mitochondria lack the enzymes needed for GSH biosynthesis, therefore the mitochondrial GSH pool must be imported from the cytoplasm [26]. This tripeptide is mainly found in mitochondria in its reduced form. It represents 10–15% of total cellular GSH, with a concentration range of 5–10 mM [31]. Glutathione cannot freely cross a lipid bilayer because it is negatively charged at physiological pH, so the outer mitochondrial membrane (OMM) and inner mitochondrial membrane (IMM) must be equipped with transporters or channels to facilitate the entry of GSH. The OMM is rich in porins that form aqueous channels through the lipid bilayer and allow diffusion between the intermembrane space (IMS) and the cytosol of molecules smaller than ~5 kDa, including glutathione [26]. Kojer demonstrated that glutathione pools in the IMS and the cytosol are linked by porins [33]. The inner membrane (IMM) is where, in mammalian cells, the dicarboxylate carrier (DIC) and the oxoglutarate carrier (OGC) were described to carry most of the GSH [34]. On the other hand, it has been reported that DIC and OGC together represent only 45–50% of the total glutathione uptake in hepatic mitochondria, so it has been proposed that the glyoxalase system contributes to mitochondrial GSH supply. This metabolic pathway is widespread in all biological systems and is involved in the cellular detoxification of α-ketoldehydes produced during glycolysis; it catalyzes the conversion of 2-oxaldehyde to 2-hydroxyacid, through the intermediate S-2-hydroxyacylglutathione. The glyoxalase system consists of two enzymes, glyoxalase I (Glo I) and glyoxalase II (Glo II) and GSH as a cofactor. In the cytosol, Glo I catalyzes the formation of S-D-Lactoylglutathione (SLG) from hemithioacetal (MeCOCH(OH)-SG) generated from methylglyoxal (MG) and GSH. The SLG can enter the mitochondria and through Glo II is hydrolyzed into D-lactate and GSH; this represents a complementary mechanism for the supply of GSH to the mitochondria [35].

The concentration of GSH present in the mitochondria is kept constant due to the transport of GSH from the cytosol, through two GSH transportation systems, one of high-affinity, stimulated by ATP, and one of low-affinity, stimulated by ATP and ADP [36]. In the case of endoplasmic reticulum, evidence suggests the presence of a transportation system that allows the selective passage of GSH onto GSSG [37]. In this organelle, GSH contributes to the reduction of protein-disulfide isomerase (PDI), responsible for catalyzing the formation of disulfide bonds in proteins [32,37]. The use of GSH to maintain oxidoreductases in their reduced form leads to a constant production of GSSG in the lumen of the endoplasmic reticulum. GSSG is transported to the cytosol with facilitation of diffusion through the Sec61 protein-conducting channel [38], where it is reduced by the enzyme glutathione reductase [31,37,38].

**Figure 2:**
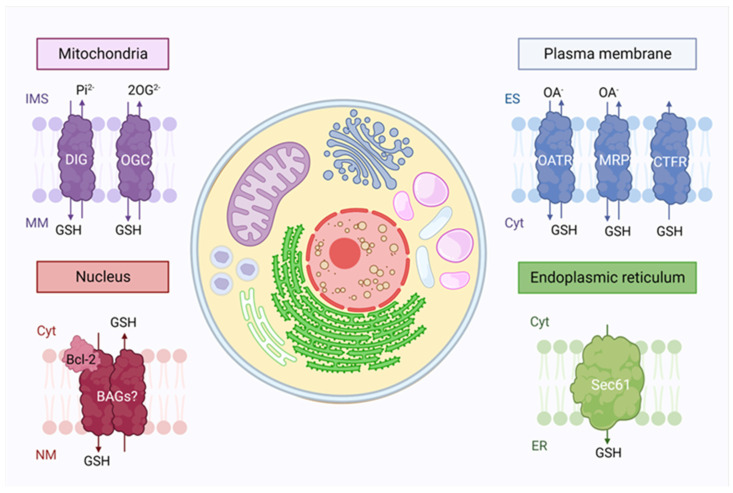
Glutathione intracellular compartmentalization. Glutathione synthesis takes place only in the cytosol (cyt), but it is distributed to many organelles due to the presence of transporters. In mitochondria, the outer membrane contains a large amount of porins, which allow glutathione transport, while dicarboxylate (DIG) and the oxoglutarate (OGC) transporters are present in the inner membrane. In the nucleus, Bcl-2 proteins are believed to be involved in the GSH translocation through Bcl2-associated athanogene pores (BAG). Glutathione is also found in the endoplasmic reticulum (ER), where its facilitated diffusion occurs through the Sec61 protein-conducting channel. Finally, the exchange between extracellular and intracellular glutathione in the plasma membrane occurs through the functioning of three families of transporters: the organic-anion-transporting polypeptide (OATR), the drug resistance-associated proteins (MRP) and cystic fibrosis transmembrane conductance regulator (CTRF). IMS: Intermembrane space, MM: Mitochondrial matrix, NM: Nuclear matrix, ES: Extracellular space.

The mechanisms of nuclear glutathione transport and sequestration are under discussion [39]. Certainly, the synthesis of GSH does not take place in the nucleus because, like mitochondria, it lacks the enzymes required for GSH biosynthesis [26]. Bcl-2 proteins possess a BH-3 domain where GSH binds and since its presence seems to be correlated to the increase of the GSH pool in the nucleus, it is possible that Bcl-2 proteins are involved in GSH translocation into the nucleus through Bcl-2 associated athanogene pores (BAG) [39,40,41]. Diaz Vivancos et al. (2010) proposed a model for the glutathione cycle in the nucleus [27]. In this model, GSH is recruited and directed to the nucleus in the early G1 phase of cellular division; thus, GSH increases in the nucleus while cytosolic GSH is depleted. The altered cytosolic redox environment promotes the synthesis of new GSH, whereby the overall glutathione pool significantly increases; the nuclear envelope dissolves so that there is a rebalancing between cytosolic and nuclear GSH during G2 and M phase. During telophase, the nuclear membrane reassembles, the cell divides and the total GSH pool is allocated equally among the daughter cells (Figure 2) [27].

The redox state of GSH/GSSG in plasma is controlled by multiple processes, including the synthesis of GSH from its constitutive amino acids, cyclic oxidation and reduction involving GSH peroxidase and GSSG reductase, protein S-glutathionylation, transport of GSH into plasma, and degradation of GSH and GSSG by γ-glutamyltranspeptidase [31,42].

GSH is present in all mammalian cells in a constant state of metabolic recirculation (synthesis, degradation, and irreversible loss of GSH). Its half-life is 4 days in human erythrocytes, 2 to 4 h in the cytosol of rat hepatic cells and 30 h in the mitochondrial lumen [43]. Many different conditions affect the intracellular GSH contents, some of them being the presence of heavy metals, high glucose concentrations, heat shock, exposure to reactive oxygen and nitrogen species including H_2_O_2_ and nitric oxide, ozone exposure, ionizing radiation, cigarette smoke [25,44,45,46]. Differences between GSH content in some mammalian cells are listed in Table 1.

## 3. S-Glutathionylation

Redox regulation of cell function often involves the conversion of reactive thiols on specific cysteine residues from reduced to oxidized forms [63]. The main types of thiol modification that have been shown to play an important redox-dependent role include protein S-glutathionylation which is produced in the cell under physiological conditions and oxidative stress, both spontaneous and enzymatic [64,65]. Under S-glutathionylation, GSH may bind to cysteinyl residues in proteins by creating reversible disulfide bonds, depending on the cysteine position and redox potential [66,67].

This post-translational modification of the protein is primarily catalyzed by glutaredoxin (Grx), which leads to enhanced or suppressed activity; it can prevent protein degradation by proteolysis or sulfhydryl overoxidation, plays a key role in cellular signaling and participates in some pathological processes, including atherosclerosis, neurodegenerative disorders, cardiovascular diseases, and several types of cancer [65,68,69,70,71]. Reports suggest that protein S-glutathionylation and Grx1 carry out a wide range of antioxidant, anti-inflammatory, and anti-apoptotic functions in the body, participating in acute and chronic inflammatory responses [65,72,73].

The glutaredoxin catalytic mechanism depends on the GSH/GSSG ratio. Under an increase in GSH/GSSG, Grx can catalyze the deglutathionylation of proteins, but under conditions of decreased GSH/GSSG ratio, Grx can catalyze S-glutathionylation of proteins. It should also be noted that not only glutaredoxin can participate in these processes [64,65].

Protein S-glutathionylation regulates the structure and function of target proteins, including actin, Ras, integrins, transcription factors (NF-κB and AKT) [74], and metabolic enzymes (GAPDH, succinate dehydrogenase, and pyruvate kinase); therefore, it is required for cellular homeostasis [71,75].

## 4. Glutathione Transporters and Associated Diseases

**Figure 3:**
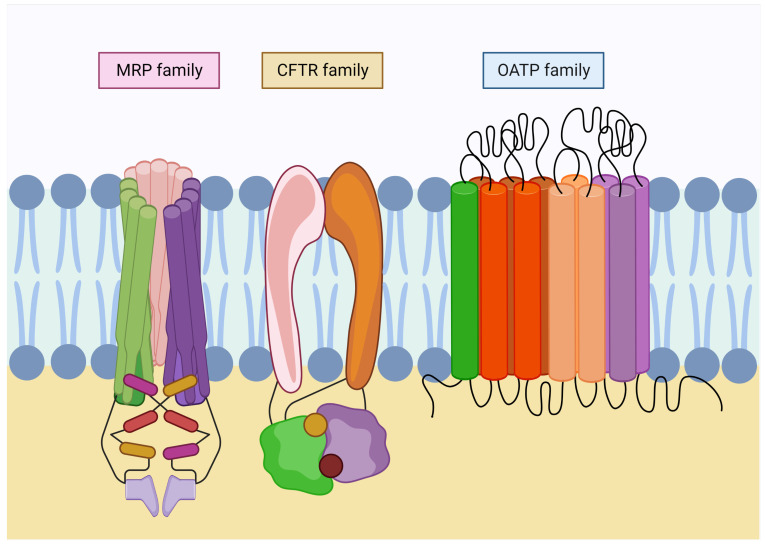
Families of glutathione transport in the plasma membrane. Glutathione transporters belong to the ABC superfamily, which requires ATP to carry out their functions. Multidrug resistance-associated proteins (MRP) are involved in GSH export and homeostasis. In addition, they conduct its derivates efflux. Moreover, the cystic fibrosis transmembrane conductance regulator family (CFTR) is involved in the export of GSH in the kidney and lungs. Finally, organic-anion-transporting polypeptide family (OATP) is widely expressed throughout the organs, acting bidirectionally and regulating the uptake of metabolites in the exchange for reduced glutathione.

There are studies that relate mitochondrial redox state and glutathione content with diseases and oxidative-induced cell death. Since GSH in mitochondria comes from the cytoplasmic reserve, the role of transporters becomes important [76]. The participation of three families of transporters in mammalian cells involved in the transportation and movement of glutathione has been demonstrated: a family of drug resistance-associated proteins (MRP), cystic fibrosis transmembrane conductance regulator family (CFTR) and organic-anion-transporting polypeptide family (OATP) (Figure 3) [77,78].

### 4.1. MRP Family

Multidrug resistance-associated proteins (MRP/ABC) are involved in GSH export and homeostasis. MRP proteins not only regulate GSH efflux, but also transport oxidized glutathione derivatives such as glutathione disulfide (GSSG), S-nitrosoglutathione (GS-NO) and glutathione-metal complexes, as well as other glutathione S-conjugates [77,79]. MRP proteins belong to the C family of the ABC transporter superfamily, which requires ATP for transportation [80]. These transporters are responsible for the movement of a wide variety of xenobiotics, including drugs, lipids and metabolic products across plasma and intracellular membranes [81]. MRPs are located in the plasma membrane of mammalian cells, while in yeast and plants they are widely located in the vacuole [76]. Moreover, the MRP family of proteins is made up of 9 transporters (MRP1-MRP9), almost all of which accept glutathione S conjugates as substrates. One of the first studies that indicated that glutathione was transported by MRP proteins was with the use of a lung carcinoma cell line that overexpresses the MRP1 protein; as a result it was found that it had lower levels of intracellular GSH and higher levels of extracellular GSH [82].

#### 4.1.1. MRP1

The first human MRP identified as a GS-X and/or GSSG transporter was the ATP-binding cassette (ABC) protein ABCC1, first known as GSH conjugate pump and later identified as multidrug resistance protein MRP1 [83,84]. MRP1 is one of the most described transporters; knowledge of its molecular mechanisms and physiological functions related to GSH transport and GSH conjugates is the most advanced of all MRP-related proteins [79].

MRP1 carries a wide variety of anticancer drugs, including but not limited to vincristine, etoposide, anthracycline, and methotrexate (MTX). MRP1 has also been shown to transport other drugs used in the treatment of non-malignant diseases, such as opioids, antidepressants, statins, and antibiotics [85]. In addition to its role in the cellular extrusion of xenobiotics, MRP1 exports other physiologically important molecules. These include proinflammatory molecules (e.g., leukotriene C4), hormones (e.g., estrogens and prostaglandins), and antioxidants (e.g., oxidized and reduced glutathione) [86].

MRP1 is involved in inflammation, detoxification, and oxidative stress. A high level of MRP1 expression was associated with poor clinical outcomes in children with neuroblastoma [87]. Overexpression of MRP1, which represents the strength of cancer cells, can be targeted by substances such as verapamil, which specifically target this transporter and trigger lethal oxidative stress in the cancer cell. MRP1, when overexpressed, has been shown to regulate basal and apoptotic GSH release, suggesting that it plays a key role in these processes [87,88]. Recently, it was also found to act as a player in ferroptosis by regulating the abundance of intracellular glutathione. MRP1 is identified as a GSSG transporter. Inhibition of MRP activity has been shown to promote the accumulation of GSSG, which is cytotoxic to endothelial cell tumors. MRP inhibition could reduce drug resistance in cancer cells, and MRP acts as a potential target in cancer therapy [89].

#### 4.1.2. MRP2

Meanwhile, the MRP2 transporter can also transport organic anions, including sulfate, glucuronide, and GSH conjugates. In addition, MRP2 is also responsible for the biliary elimination of certain endogenous conjugates, such as leukotriene-C4 (LTC4) and conjugated bilirubin [90].

Mutations in the MRP2 gene are associated with Dubin–Johnson syndrome, a condition due to the lack of hepatobiliary transport of organic anions without bile salts resulting in conjugated hyperbilirubinemia [81,82]. Studies have demonstrated that this transporter is one of the ABC pumps with the highest-level of expression in organs important for endo- and xenobiotic metabolism, such as the liver, kidneys, and intestine [81]. The MRP2 transporter is known to be present in some malignant human tumors, as demonstrated by immunostaining of hepatocellular, clear cell renal, colorectal, ovarian, leukemia, mesothelioma, lung, breast, bladder, and gastric cancer samples [83].

#### 4.1.3. MRP3

MRP3 is a relatively poor transporter of GSH-conjugated organic anions compared to MRP1 and MRP2 [91]. In humans, MRP3 is primarily expressed in the adrenal glands, kidney, small intestine, colon, pancreas, and gallbladder, with a lower magnitude of expression in the lungs and spleen. MRP3 appears to play a compensatory role in the loss of MRP2 in the liver. Elevated levels of MRP3 expression have been detected in human hepatocellular carcinomas, primary ovarian cancer, and adult acute lymphoblastic leukemia. Furthermore, MRP3 overexpression was predicted to be a prognostic factor in childhood and adult acute lymphoblastic leukemia and adult acute myeloid leukemia [90].

#### 4.1.4. MRP4

MRP4 is a widely versatile transporter that exhibits high substrate specificity composed of a wide variety of amphipathic anions, including steroid and eicosanoid conjugates, as well as cyclic nucleotides and nucleotide analogs. MRP4 has been shown to play a role in cyclic adenosine monophosphate (cAMP) homeostasis in vascular smooth cells and cardiac myocytes [92]. Other tasks have been proposed for MRP4 in platelets, considering that its location can shift from the granules to the plasma membrane when platelets are activated and under certain pathophysiological conditions [93]. These include the release of lipid mediators, as well as a role in aspirin resistance under certain conditions, such as in patients after coronary artery bypass graft surgery [94].

In addition to its localization in the plasma membrane, MRP4 was shown to be found in large amounts in the membrane of dense granules. An altered distribution of MRP4 was observed in platelets from a patient with Hermansky–Pudlak syndrome, in which MRP4 was only detected in the plasma membrane due to the lack of dense granules [95].

#### 4.1.5. MRP5

MRP5 was identified as transporting cAMP, cyclic guanosine monophosphate (cGMP), and antiretroviral compound PMEA (9-(2-phosphonomethoxyethyl)adenine). It has also been shown to transport nucleotide/nucleoside analogues and GSH conjugates [96]. MRP5 expression was widely detected in human tissues such as liver, placenta, and cornea, and in carcinomas. MRP5 expression level was also associated with cisplatin exposure. Several in vitro studies suggested that MRP5 would transport several anticancer drugs, including MTX, purine and pyrimidine analogs [97].

#### 4.1.6. MRP6

MRP6 is an organic anion transporter that is mainly distributed on the basolateral side of hepatocytes and on the proximal tubules of the kidney [98]. Although MRP6 is not involved in drug resistance, it may be a constitutive transporter in normal and abnormal hepatocytes [90]. MRP6 can also mediate the transport of glutathione conjugates, LTC4 and N-ethylmaleimide S-glutathione (NEM-GS) [99].

Mutations found in the MRP6 gene are associated with genetic abnormalities of the autosomal inherited connective tissue disorder called pseudoxanthoma elasticum (PXE), which is characterized by the presence of dystrophic elastic fibers in the skin, retina, and large blood vessels, causing the appearance of bags in the skin, loss of vision and calcification of blood vessels [100].

#### 4.1.7. MRP7

MRP7 is a lipophilic anion transporter found primarily in the heart, liver, skeletal muscle, and kidney. MRP7 has a similar substrate range to MRP1-MRP4 and is involved in phase III (cell extrusion) of detoxification [101], but MRP7 does not engage in direct GSH transportation [102].

#### 4.1.8. MRP8

MRP8 is an amphipathic anion transporter that is functional for the efflux of purine and pyrimidine nucleotide analogs including cAMP and cGMP, and may also transport GSH conjugates [103]. MRP8 is widely expressed in the human body, with the highest levels in the liver, brain, placenta, breasts, and testis [99]. Although there is a report showing a decrease in MRP8 level in breast cancer, a high level of MRP8 was reported in breast cancer and gastric cancer cell lines [96].

#### 4.1.9. MRP9

MRP9 does not transport typical substrates such as drug conjugates and other substances as do other MRP members [104]. It is highly expressed in breast cancer, normal breast, and testis; however, its functions are still unknown [105]. There is a study showing that in the joint absence of MRP5 and MRP9, some metabolites such as heme and some others are poorly transported or distributed, causing mitochondrial damage [106].

The available information for this family of transporters is summarized in Table 2.

### 4.2. Family CFTR

CFTR proteins belong to the C family of the ABC transporter superfamily [80]. CFTR is best known as a chloride channel, but it has also been shown to facilitate GSH export in kidney cell lines and lung tissue [118].

The absence of functional CFTR disrupts epithelial water and ion homeostasis, leading to the accumulation of dehydrated mucus, recurrent bacterial infections, and ultimately organ failure and other life-threatening consequences [78]. Cystic fibrosis primarily affects the lungs, but also affects the pancreas, intestine, liver, kidneys, and sweat glands [119]. For example, a study in 16-HBE bronchial epithelial cells showed that CFTR gene expression is increased after 48 h of exposure to cigarette smoke, demonstrating that CFTR expression can be induced. It is possible that CFTR expression decreases as an initial response, but as exposure time increases, and as an adaptive antioxidant response, CFTR expression is induced [120]. However, in a previous study carried out in Calu-3 cells, it was shown that exposure to cigarette smoke causes a decrease in the synthesis of CFTR mRNA, which was reflected in the expression of the protein [121].

Another study demonstrated that CFTR deficiency occurs in the nasal respiratory epithelium of smokers [120]. One of the most likely causes of the decreased function would be the increase in heavy metals found in cigarette smoke, particularly cadmium, which has been shown to inactivate CFTR [122].

One report from 2005 indicates that human CFTR channels are reversibly inhibited by several reactive forms of glutathione (i.e., glutathione disulfide S-oxide [GS(O)SG], nitrosylated glutathione [GSNO] and GSSG), and glutathione treated with diamide, a strong thiol oxidizer. The underlying mechanism appears to involve the glutathionylation of cys-1344 near the signature sequence in the cytoplasmic nucleotide binding domains (NBDs); this region is predicted to participate in ATP-dependent channel opening [123,124]. Channels could be protected from inhibition by pretreating with *N*-ethylmaleimide (NEM) a thiol alkylating agent, or by reducing agents such as dithiothreitol (DTT) or by the actions of GSH and glutaredoxin [124]. This finding is important because the CFTR channel is expressed in the lung and gut; these tissues are continuously exposed to thiol oxidants under a variety of inflammatory conditions, allowing the reactive glutathione species that are formed to have the potential for glutathionylation of these channels [125].

### 4.3. OATP Family

The family of organic-anion-transporting polypeptides (OATPs) consists of eleven human OATPs, which are classified into six different OATP1–6 subfamilies. OATP1 is mainly found in human hepatocytes [126]. However, its expression has been demonstrated in different tissues such as the blood-brain barrier (BBB), choroid plexus, lung, heart, intestine, kidney, placenta, and testis [127]. The three OATPs that are most abundantly expressed in the liver are OATP1B1, OATP1B3 and OATP2B1. These transporters act bidirectionally and regulate the uptake of amphipathic and anionic substances in exchange for reduced glutathione or bicarbonate [128].

The OATP family of transporters functions independently of ATP and sodium gradients, and instead, relies on the large GSH electrochemical gradient across the plasma membrane. Two members of the OATP family, OATP1 and OATP2, have been shown to mediate GSH export by exchanging GSH for solute uptake.

OATP1A1 has been found in cell types other than the proximal tubules, such as hepatocytes, which use the GSH electrochemical gradient to drive organic anion uptake [129].

There have been no studies showing that lack of OATP function causes disease. However, in several models of cholestatic liver diseases, such as endotoxin treatment, ethinylestradiol treatment and bile duct ligation, the expression of hepatocellular OATPs was down-regulated [130].

It is well known that malignant cell transformation alters the pattern of OATP expression in organs. In fact, the gonad specific OATP6A1 has been identified as a carcinogenic antigen in lung tumors and lung tumor cell lines [131]. Human Rotor syndrome is an inherited disorder associated with OATPs. It is an autosomal recessive disorder characterized by conjugated hyperbilirubinemia, coproporphyrinuria and almost no hepatic uptake of anionic diagnostic agents due to genetic variants in OATP1B1 and OATP1B3 [132].

## 5. Glutathione Deficiency Causes

The alteration of glutathione transport activity is related to the deficiency or low activity of the transporters mentioned in the previous section, which is reflected in an increase in intracellular glutathione concentrations. This condition is associated with some pathologies [87,88,119].

In humans, a decrease in GSH has been associated with different conditions, such as deficiency of the enzymes involved in glutathione synthesis [133]; in this case, individuals show a limited or generalized deficiency of GSH and an accumulation of 5-oxoproline (in blood and cerebrospinal fluid) leading to metabolic acidosis [43], mental retardation, neuropsychiatric dysfunction, spinocerebellar degeneration, peripheral neuropathy, myopathy, hepato-splenomegaly, hemolytic anemia, aminoaciduria, and severe neurological complications [134]. These individuals may also develop hypersensitivity to antibiotics and are more prone to influenza virus infections [43].

For the aforementioned reasons, the maintenance of high concentrations of GSH is vital for most types of cell, since it plays important roles in the control of biological process, including metabolic detoxification, protein folding, vitamin regeneration, mitochondrial health, immune defense against viruses, cellular proliferation regulation, apoptosis, and redox balance, among others. Control of GSH levels is a proposed strategy for health improvement and disease prevention [135,136].

Altered levels of GSH could be the result of defects in the enzymes involved in its metabolism [133] and its excretion through the plasma membrane [137]. An example is deficiency of CFRT, a protein involved in GSH transmembrane transportation, resulting in a decrease in the GSH efflux, reducing its extracellular availability [138] and inducing an oxidative state, which ends in the apoptosis process [31,138,139].

Another cause is the deficiency of enzymes related to the reduction of GSSG to GSH. For example, in erythrocytes, a deficiency of glucose-6-phosphate dehydrogenase (G6PDH) generates a decrease in the concentration of NADPH, necessary for the regeneration of GSH. This contributes to a decrease in the intracellular content of GSH and therefore, less is exported to the outside of the cell [134]. Furthermore, stress-promoting exogenous agents, for example, smoking [140], acetaminophen consumption [141] bacterial and viral infections, alcoholism, excessive exercise, emotional stress, X-ray, or sun ultraviolet light exposure [133,141] could alter GSH levels due to the amount of ROS generated. Moreover, age also influences the loss of GSH, even in healthy individuals, yet antioxidant defenses decrease [133].

**Figure 4:**
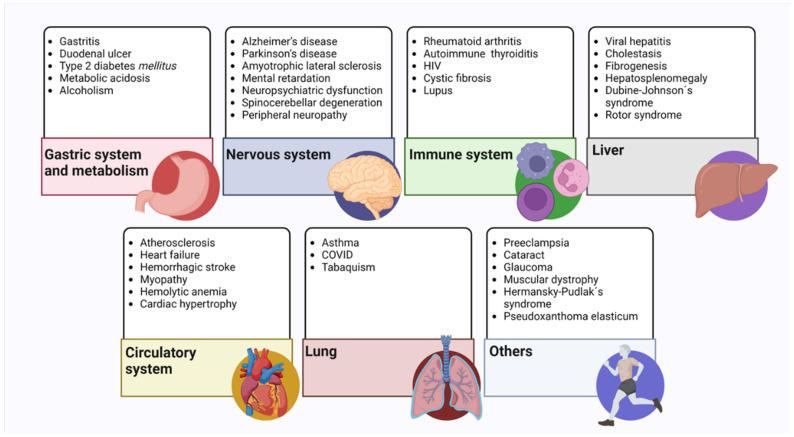
Pathologies related to the decrease in glutathione levels. These are mainly illnesses that cause a decrease in endogenous glutathione levels. All the pathologies mentioned in the text are classified according to the system or organ involved [81,82,87,95,96,98,119,130,132,134,148,149,150,151,152].

**Figure 5:**
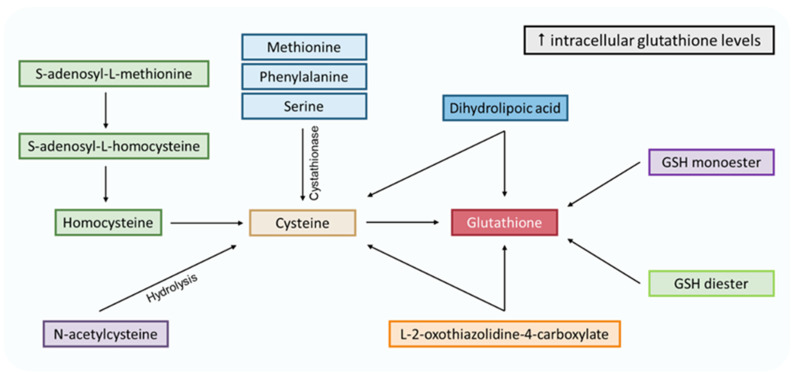
Pathways to increase intracellular levels of glutathione. Methionine, phenylalanine, serine and S-adenosyl-L-methionine are cysteine precursors through the transsulfuration pathway. N-acetylcysteine could be transformed into cysteine via hydrolysis. L-2-oxothiazolidine-4-carboxylate is an analog of 5-oxoproline (glutamate cycle form), which increases cysteine and glutathione intracellular levels. Dihydrolipoic acid comes from the α-lipoic acid reduction and can reduce glutathione and cysteine. The latter enters the γ-glutamyl cycle, where it stimulates glutathione synthesis. Finally, the GSH monoester and diester can be hydrolyzed to GSH.

Many other pathologies result in a decrease of GSH levels (Figure 4). The reasons are diverse, but most coincide with defects in the synthesis or transportation of enzymes or a shortage of precursors. The organism uses different pathways to successfully increase the intracellular levels of glutathione and during exogenous regulation, the involved reactions are summarized in Figure 5 [142,143,144,145,146,147].

## 6. Glutathione and Disease

Plasma glutathione GSH and GSSG levels vary, depending on the life span of a healthy individual (see Table 3). However, these concentrations can vary significantly when a disease generates and maintains oxidative stress for long periods, which would produce a decrease in the concentration of GSH and an increase in GSSG. Below, we describe some diseases that increase oxidative stress and thereby considerably affect the recovery time of patients.

### 6.1. Cardiovascular Disease (CVD)

An imbalance in redox homeostasis could cause cardiovascular complications. Development and progression of CVD have been characterized by changes in the concentration of GSH or its oxidation state [20]. There are some mechanisms involved in GSH diminution: increased oxidation by intracellular oxidizing agents, increased conjugation to molecules, and increased exit across the cell membrane [137].

Many animal studies have demonstrated the role of GSH in CVD. For example, heat shock proteins (HSPs) have shown protection against several stress stimuli in mammalian cells. Human heat shock protein 27 (Hsp27) and murine heat shock protein 25 (Hsp25) protect against H_2_O_2_ by increasing levels of reduced GSH in a G6PDH-dependent manner [156]. Furthermore, degradation of nuclear factor erythroid 2–related factor 2 (Nrf2) has been found to contribute to the decreased expression of several antioxidant enzymes [157]. In addition, Nox4 facilitates cardiac-related adaptation to chronic stress by activating Nrf2, which increases concentrations of GSH and, consequently, increases the GSH/GSSG ratio [158]. In serum from atherosclerotic mice or mouse models with apolipoprotein E deficiency, GSH, transported in liposomes, reduced susceptibility to oxidation by 2,2′-azobis(2-amidinopropane) dihydrochloride (AAPH). GSH levels in peritoneal macrophages increased in these mice, but lipid peroxides and oxidized LDL levels decreased [159]. Another research study found that N-acetyl cysteine (NAC) might boost GSH levels and reduce liver and plasma cholesterol levels in mice fed a high-fat diet [160]. Moreover, in the process of atherogenesis, the ability of macrophages to synthesize glutathione is inversely related to the initiation and progression of atherosclerosis in apolipoprotein E deficient mice (Apo E-/-). Oxidative stress is an important factor in atherogenesis. Under oxidative stress, lipid peroxidation is observed in LDL and arterial wall cells, leading to the formation of atheromatous plaques. Macrophage GSH decreases the cellular oxidative state, the ability of macrophages to oxidize LDL, and the development of atherosclerotic lesions in Apo E-/- mice [161]. Again, glutathione peroxidase 1 (GPx-1) is a critical enzyme in the protection of vessels against atherogenesis. In the diabetic apolipoprotein E-deficient mouse model, decreased levels or lack of GPx-1 accelerate diabetes-associated atherosclerosis [162,163].

Furthermore, several human studies demonstrate that GSH has a positive effect on the cardiovascular system. According to several studies, patients with heart disease and diabetes have reduced plasma GSH levels. Additionally, patients with CVD have lower GSH levels than subjects without a CVD history [164]. Type 2 diabetes mellitus (T2DM) patients showed decreased levels of GSH, and of enzymes involved in GSH synthesis. In other studies, GSSG and transforming growth factor-beta (TGF-β) levels were higher in diabetic patients. In this case, an increased level of proinflammatory cytokines and a decreased expression of enzymes involved in GSH synthesis were observed [150]. The increased level of GSH in plasma leads to reduced values of systolic and diastolic pressure and a decreased incidence of diabetes [164]. In addition, the levels of GSH and GSSG were measured in mononuclear cells in hypertensive patients with or without different antihypertensive therapies. In hypertensive patients, the levels of GSH decreased while the levels of GSSG increased. Three months of antihypertensive treatment reduced oxidative stress and GSSG and increased the levels of GSH [165].

Plasma GSH, on the other hand, decreased by 21% and 40% in patients with asymptomatic and symptomatic CVD, respectively. These results indicate that decreases in the level of GSH are related to cardiac abnormalities in patients with CVD [166]. The blood test to measure the level of GSH should be used as a new biomarker to detect CVD in asymptomatic patients [166]. Additionally, increased oxidative stress could lead to myocardial infarction (MI) in cardiac procedures. The glutathione S-transferase (GST) polymorphism has been identified as a factor that could increase MI in cardiac surgery. Decreased GPx-1 activity increases risks of stroke and coronary heart disease. Thus, measuring erythrocyte GPx-1 levels might be used as a predictive value, and increasing the level of GPx-1 could have a beneficial effect on CVS. Furthermore, studies in patients with T2DM suggest that GPx-1 is an essential enzyme that plays a protective role in the development of endothelial dysfunction and atherosclerosis in diabetes [149]. The effect of antioxidants in patients with atherosclerosis has also been studied in humans. The results demonstrated that administrating NAC increased GSH levels and reduced endothelial adhesion molecule levels, potentially preventing vascular damage in diabetic patients. These results showed how glutathione has antioxidative and antiatherogenic properties and can lead to the remission of atherosclerosis [167].

Accordingly, treatment with GSH could reduce oxidative stress and prevent related diseases. However, the administration of GSH would not be the best solution because intestinal and hepatic gamma-glutamyl transferase (GGT) metabolizes GSH and decreases the level of administered GSH [168,169]. Therefore, the administration of pure GSH in the form of an orobuccal fast-slow-release tablet on healthy volunteers has been evaluated. In this trial, it was observed that an increased level of GSH in the blood could result from GSH absorption through mouth mucosa [170]. Other researchers compared the level of GSH and other oxidative stress markers in the blood of subjects with metabolic syndrome after administration of different forms of GSH (oral and sublingual) and NAC [171].

### 6.2. Neurodegenerative Diseases

Neurodegenerative diseases, such as Parkinson’s (PD), Alzheimer’s (AD), amyotrophic lateral sclerosis (ALS), and Huntington’s, share several common features in pathogenesis, such as (i) the accumulation of abnormally aggregated proteins (pathological inclusions), (ii) oxidative damage and (iii) mitochondrial dysfunction [172]. Each condition causes alterations in different pathways that enable oxidative damage to establish itself. Below is a brief description of these changes in some of the most common neurodegenerative diseases.

#### 6.2.1. Alzheimer’s Disease (AD)

AD is pathologically characterized by amyloid β (Aβ) deposition and neurofibrillary tangles in the brain, and loss of synaptic connections in specific areas of the brain [173]. Nuclear factor erythroid 2-related factor 2 (Nrf2) is a crucial redox-regulated gene in controlling ROS levels; as intranuclear Nrf2 is decreased in AD, this causes the accumulation of ROS, senescent organelles, and misfolded proteins [174]. The regulation of the expression of excitatory amino acid carrier 1 (EAAC1) is promoted by Nrf2. EACC1 is the regulatory mechanism of neuronal GSH production; therefore, by decreasing the expression of Nrf2, the expression of the EAAC1 protein is suppressed, which leads to a decrease in cysteine uptake and consequently to decreased brain GSH levels, and vulnerability to oxidative stress [174,175].

#### 6.2.2. Parkinson’s Disease (PD)

Neuronal loss in the substantia nigra (SN) is a neuropathological characteristic of PD, which leads to striatal dopaminergic insufficiency and an increase in the synthesis of α-synuclein in neuronal inclusions [176]. The α-synuclein binds to ubiquitin and forms cytoplasmic inclusions of proteins called Lewy bodies; α-synuclein could induce generation of abundant ROS and inflammatory factors, causing lipid peroxidation and death of neurons [177]. This causes a decrease in the level of GSH, which constitutes the main antioxidant defense of dopaminergic neurons [178]. As with AD, the Nrf2 is the main protein involved in the development of ROS-caused PD [177].

#### 6.2.3. Amyotrophic Lateral Sclerosis (ALS)

ALS is a progressive, fatal neuromuscular disorder characterized by the degeneration of upper and lower motor neurons leading to somatic muscle dysfunction in the body [179]. In 90% of hereditary cases of ALS, patients have mutations in the superoxide dismutase 1 (SOD1) enzyme which converts O_2_•− to H_2_O_2_ and O_2_ to protect cells from ROS, and is directly associated with oxidative stress and inflammation [180]. Recent clinical studies showed that GSH levels in the brains of ALS patients were decreased compared to those of age-matched healthy volunteers [181], and the decrease of GSH levels was more prominent in the motor cortex than in the white matter of ALS patients [182]. These results suggest that the brains of patients with ALS have limited antioxidant capacity [180].

#### 6.2.4. Huntington’s Disease (HD)

This disease is characterized by an increase in the number of repeats of the cytosine, adenine, and guanine (CAG) triplet in the Huntington gene, located on the short arm of chromosome 4, which codes for a protein rich in glutamine residues known as huntingtin (HTT); therefore, this is considered a hereditary disease with an autosomal dominant pattern [183]. Some of the indicators of oxidative damage that have been observed in the striatum and cerebral cortex of patients with HD are an increase in the concentration of malondialdehyde (MDA) and 4-hydroxynonenal (lipid oxidation products), increase in carbonylation and in protein nitration, as well as a decrease in GSH and an increase in the activity of glutathione peroxidase, catalase, and superoxide dismutase [184].

### 6.3. Diabetes Mellitus Type 2

Oxidative stress contributes to the pathogenesis of diabetes mellitus type 2 (DM2) by increasing insulin resistance or affecting insulin secretion [185]. Hyperglycemia increases free radical production and impairs the antioxidant defense system [186]. In patients with DM2, there is a decrease in antioxidant defenses, together with reduced levels of specific antioxidants such as ascorbic acid and vitamin E, and decreased activity of antioxidant enzymes such as catalase, superoxide dismutase and glutathione peroxidase [187]. In addition, antioxidant vitamins such as ascorbic acid and tocopherols have been reported to improve insulin sensitivity [188]. Patients with DM2 have a reduced level of GSH, high levels of GSSG and TGF-β, increased levels of proinflammatory cytokines, and decreased expression of enzymes involved in GSH synthesis. Oral GSH supplementation improves insulin sensitivity, reduces oxidative stress levels, and prevents GSH depletion in individuals with DM2. GSH supplementation also increases the levels of Th1-associated cytokines, IFN-γ, TNF-α, and IL-2, and decreases the levels of proinflammatory cytokines such as IL-6 and IL-10 in these individuals [189]. Reduced GSH concentration levels in DM2 patients’ red blood cells, plasma, and monocytes are accompanied by decreased expressions of glutamate cysteine ligase (GCL), GSH synthetase (GS), and gamma-glutamyl transpeptidase (GGT), and a decreased substrate, since cysteine and glycine supplementation partially restore the GSH concentration in these patients [190]. Sodium tungstate is an alternative to reduce hyperglycemia in the treatment of diabetes. The reduction of hyperglycemia by sodium tungstate reduces lipid peroxidation and causes alterations in the antioxidant system in the salivary glands of diabetic rats induced by streptozotocin (STZ) increasing the GSH/GSSG ratio [191].

On the other hand, both in animal models and in patients, DM2 is frequently accompanied by islet fibrosis. Several in vivo and in vitro studies have shown that antioxidants can successfully inhibit pancreatic fibrosis. GSH can inhibit the activation and proliferation of pancreatic stellar cells (PSCs), thereby inhibiting pancreatic fibrosis and protecting islet β cells from damage [192].

### 6.4. Cancer

ROS are important in the processes of growth, proliferation, metastasis, and survival of tumor cells. These cells have higher levels of ROS and greater expression and activity of antioxidant systems than non-cancerous cells. Upregulation of NRF2 (nuclear factor, erythroid-derived 2-like factor 2) and elevated GSH levels have been observed in various tumors, including breast, ovarian, prostate, skin, lung, and pancreatic tumors [193]. NRF2 regulates the expression of several enzymes responsible for glutathione synthesis [194]. High ROS production in cancer cells requires high activity of cellular antioxidant systems, making cancer cells hypersensitive to agents that impair their antioxidant capacity. Therefore, the reduction in the production or availability of GSH may be important for tumor therapy [195]. Additionally, GSH acts as a detoxifying agent, so in cancer cells, this process can be used for the removal of chemotherapeutic drugs. Thus, GSH plays an important role in chemotherapy resistance, and its inhibition as part of combination therapies has been shown to be an important approach to improve the efficacy of chemotherapies [196]. On the other hand, because of the high concentration of GSH in many tumors and its high reactivity, GSH is used as an activator of prodrugs, such as romidepsin, which is used to treat cutaneous T-cell lymphoma and other peripheral T-cell lymphomas [197]. In addition, the development of ROS- and GSH-sensitive nanoparticle drug delivery systems has been proposed to deliver a highly toxic load more specifically and safely to cancer cells. The GSH-induced disintegration of nanoparticles was demonstrated as an example of this system, allowing the release of active platinum metabolites, which covalently bound to the target DNA and induced apoptosis in cancer cells; however, this technology requires further investigation for optimization before clinical use [198]. Moreover, cancer cells that are resistant to radiation and chemotherapy have elevated GSH levels, probably due to GSH’s ability to quench the ROS generated by these therapies [199].

Beyond its role in cancer cells, GSH synthesis has implications in the tumor surrounding microenvironment (TME) for non-malignant cells. For example, obesity is a risk factor for numerous malignancies and may promote tumorigenesis. Lowering GSH levels, either genetically or pharmacologically, prevents obesity induced by a high-fat diet. Therefore, GSH could promote lipid accumulation or storage and support a TME that favors tumor growth [200,201]. Another group of cells that have been implicated in GSH metabolism are immune cells. T cells show similar dependence on GSH as cancer cells due to ROS increase during periods of proliferation. Deletion of proteins important for GSH synthesis in T cells leads to an altered immune response, suggesting that GSH is required for antitumor immunity [202].

### 6.5. COVID-19

It has been reported that patients infected with COVID-19 disease have higher oxidative/nitrosative stress and a substantial decrease in vitamin D, as well as alterations in thiol levels, total antioxidant capacity, GSH and selenium. This appears to be a common pathway related to the high mortality from COVID-19 [152,203]. It was also found that in Covid patients, there was a decrease not only in GSH levels but also in vitamins such as A, C and E, as well as enzymes that combat oxidative stress such as glutathione peroxidase, superoxidodismutase and catalase [204].

GSH deficiencies have been found in people hospitalized with COVID-19, particularly in younger humans. This is a significant finding because younger humans are not expected to be GSH deficient [205]. Furthermore, it has been reported that even children with COVID-19 showed this GSH deficiency when compared to control values [206]. Finally, it is known that this deficiency depended on age and was more pronounced in older people. In addition, in patients with COVID-19, increased lipid peroxidation and damage due to oxidative stress were observed. Compared to control samples, significantly reduced levels of GSH were observed in postmortem cortical samples from COVID-19 patients. SARS-CoV-2 also induced oxidative stress-mediated changes in the testes and epididymis, as seen from COVID-19 postmortem autopsies compared with controls. GSH levels decreased with increasing severity of COVID-19 [207].

## 7. Conclusions

Glutathione plays an important role in antioxidant defense and in the regulation of the pathways necessary for cellular homeostasis, not only as a detoxifier of endogenous and exogenous compounds, but also through its participation in processes related to the modulation of the synthesis of DNA, gene expression, cell proliferation, apoptosis, S-glutathionylation of proteins, signal transduction, regulation of the immune system and metabolism of cellular compounds, among others.

Furthermore, glutathione is important for the proper functioning of the metabolism, considering its cellular distribution and transport. Glutathione transporters are particularly essential because they minimize fluctuations in its concentration, as well as regulating the redox state of glutathione in different cellular compartments, while their synthesis, degradation, and recycling functions act in a coordinated manner.

Finally, glutathione deficiency is known to contribute to oxidative stress, and has an important role in aging, as well as in the pathogenesis of different diseases, such as neurodegenerative diseases, liver and kidney disorders, cystic fibrosis, diabetes, and cardiovascular illness. In any case, it is desirable to maintain an optimal state (concentrations and redox state) of this cellular tripeptide. As described in this review article, different glutathione forms are present in the cell, all of which have specific and important functions. All this makes the study of this small tripeptide even more interesting. Although a wealth of information exists about glutathione, more remains to be discovered about its role in cellular regulation. Therefore, the study of glutathione is an important and extensive field of research that demands further examination to develop new prevention strategies and even therapies for many age-related diseases.

## Figures and Tables

**Table 1:** Glutathione distribution and homeostasis in different cell types.

Cell Type	GSH Cytosolic Concentration	GSH Homeostasis	References
Astrocytes	8–10 mM	Generate GSH conjugates exported from the cells by MRPs. Protect brain cells from ROS and xenobiotics	[47,48]
Neurons	0.2–2 mM	Lack of cystine transportation system, synthesis depends on cystine uptake via the cystine/glutamate exchange transporter	[49,50]
Hepatocytes	5–10 mM	Synthesis of GSH protects against oxidative stress, about 10% of total cytosolic GSH is transported to mitochondria	[51,52,53,54]
Erythrocytes	2.3–3 mM	Its levels are influenced by the environment. In addition, erythrocytes have the enzymatic machinery for the synthesis of GSH and the release of its derivates	[55,56,57]
Pneumocyte	400 μM in epithelial lining fluid	GSH protects lungs against oxidative damage. Type II pneumocytes contain more γ-glutamyl transferase than type I	[58,59,60]
Cardiomyocyte	2 mM	The insulin-signaling cascade regulates GSH concentration in ventricular myocytes by PI 3-kinase and MAP kinase pathways for controlling redox state	[61,62]

**Table 2:** Family of MRP transporters and the molecules they transport.

Transporter	Endogenous Substrates	References
MRP1	GSH conjugates, cysteinyl leukotrienes, glucuronic acid conjugates, bilirubin, estradiol, sulfate conjugates, bile salts, sulfated steroids, GSH, GSSG	[82,107,108]
MRP2	GSH conjugates, cysteinyl leukotrienes, glucuronic acid conjugates, bilirubin, estradiol, sulfate conjugates, bile salts, sulfated steroids, GSH, GSSG	[82,107,109,110]
MRP3	GSH conjugates, cysteinyl leukotrienes, glucuronic acid conjugates, bilirubin, estradiol, sulfate conjugates, bile salts	[82,107,111]
MRP4	GSH conjugates, cysteinyl leukotrienes, glucuronic acid conjugates, estradiol, sulfate conjugates, sulfate conjugates, cyclic nucleotides, bile salts	[82,111,112,113]
MRP5	GSH conjugates, glucuronic acid conjugates, cyclic nucleotides, GSH	[82,111,114]
MRP6	GSH conjugates, cysteinyl leukotrienes	[82,115]
MRP7	GSH conjugates, cysteinyl leukotrienes, glucuronic acid conjugates, estradiol	[82,116]
MRP8	GSH conjugates, cysteinyl leukotrienes, glucuronic acid conjugates, estradiol, sulfate conjugates, cyclic nucleotides, GSH	[82,116]
MRP9	Unknown, but not drug conjugates or other organic anions	[117]

**Table 3:** GSH, GSSG concentrations, GSH:GSSG ratio and EhGSH/GSSG at different stages of an individual’s life.

Life Period	GSH	GSSG	GSH/GSSG	E_h_ GSH/GSSG	Refs.
Childhood	2.7 ± 0.17 mM	0.16 ± 0.02 mM	16.8	−200–220 mV	[153,154]
Maturity	2.8 ± 0.9 mM	0.14 ± 0.4 mM	20	−200–240 mV	[154,155]
Old age	2.2 ± 2.0 mM	0.15 ± 0.03 mM	14.7	−200–240 mV	[154,155]

## Data Availability

Not applicable.
